# Rational Modulation of Liquid–Liquid Phase Separation Offers Novel Ways to Combat Tauopathies

**DOI:** 10.3390/ijms26146709

**Published:** 2025-07-12

**Authors:** Xingxing Zhang, Lumiao Wang, Nixin Lin, Meng Gao, Yongqi Huang

**Affiliations:** 1 Hubei Key Laboratory of Industrial Microbiology, Hubei University of Technology, Wuhan 430068, China; 2 Cooperative Innovation Center of Industrial Fermentation (Ministry of Education & Hubei Province), Hubei University of Technology, Wuhan 430068, China; 3 Key Laboratory of Industrial Fermentation (Ministry of Education), Hubei University of Technology, Wuhan 430068, China

**Keywords:** tau protein, liquid–liquid phase separation, liquid-to-solid transition, aggregation, neurodegenerative diseases

## Abstract

The microtubule-associated protein tau plays an essential role in regulating the dynamic assembly of microtubules and is implicated in axonal elongation and maturation, axonal transport, synaptic plasticity regulation, and genetic stability maintenance. Nevertheless, the assembly of tau into neurofibrillary tangles in neurons is a pathological hallmark of a group of neurodegenerative diseases known as tauopathies. Despite enormous efforts and rapid advancements in the field, effective treatment remains lacking for these diseases. In this review, we provide an overview of the structure and phase transition of tau protein. In particular, we focus on the involvement of liquid–liquid phase separation in the biology and pathology of tau. We then discuss several potential strategies for combating tauopathies in the context of phase separation: (i) modulating the formation of tau condensates, (ii) delaying the liquid-to-solid transition of tau condensates, (iii) reducing the enrichment of aggregation-prone species into tau condensates, and (iv) suppressing abnormal post-translational modifications on tau inside condensates. Deciphering the structure–activity relationship of tau phase transition modulators and uncovering the conformational changes in tau during phase transitions will aid in developing therapeutic agents targeting tau in the context of phase separation.

## 1. Introduction

**Figure 1:**
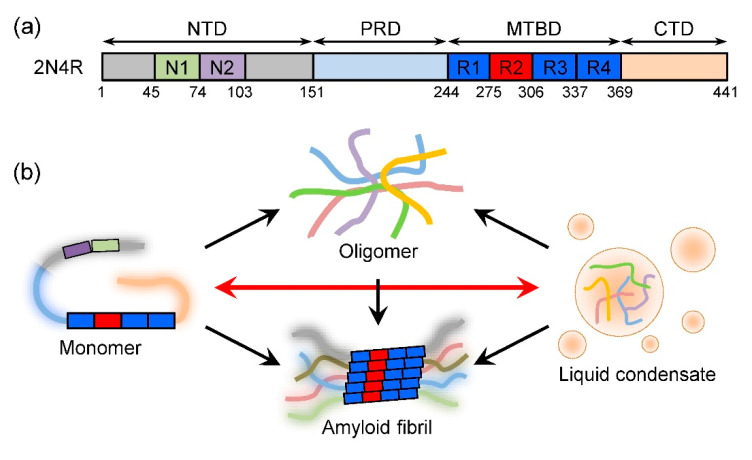
Schematic illustration of tau domain organization and phase transition. (**a**) Domain organization of 2N4R tau isoform. (**b**) Transitions among tau monomer, liquid condensate, oligomer, and amyloid fibril. The transition between tau monomer and liquid condensate is dynamic and reversible.

The microtubule-associated protein tau is a multi-function neuronal protein. It plays an essential role in regulating the dynamic assembly of microtubules [1,2]. Moreover, tau is implicated in diverse physiological processes, including axonal elongation and maturation, axonal transport, synaptic plasticity regulation, and genetic stability maintenance [2,3,4]. Nevertheless, tau dysfunction is closely associated with many diseases [2,3,4]. In particular, tau is the major component of intracellular neurofibrillary tangles (NFTs) [5,6,7], the formation of which is a pathological hallmark of a group of neurodegenerative diseases known as tauopathies [8]. Toxic tau species can transmit intracellularly and intercellularly [9,10], which induces the accumulation of tau inclusions via a prion-like cascade [11,12,13,14]. In the past decade, it has been revealed that tau can form liquid condensates via liquid–liquid phase separation (LLPS) in vitro and in cells [15,16,17,18,19,20]. Liquid tau condensates are involved in the assembly and regulation of microtubule function [21,22,23]. However, the aging of tau condensates can cause the formation of toxic tau oligomers or aggregates (Figure 1) [24,25,26,27]. These findings indicate that LLPS plays a crucial role in tau physiology and pathology [17,18].

Significant efforts have been devoted to investigating the roles of tau in neurodegeneration, suggesting that therapeutic benefits can be achieved by preventing tau aggregation and disassembling tau aggregates [28,29,30]. Meanwhile, a growing body of evidence indicates the implication of LLPS in signal transduction [31], protein homeostasis [32], post-translational modifications (PTMs) [33], and conformational dynamics [34], which offers new angles to dissect the physiology and pathology of tau. In this review, we summarize recent advances in tau structural characterization and phase transition and discuss new insights from LLPS to combat tauopathies.

## 2. Tau Protein and Tauopathies

### 2.1. Tau Physiology

The human protein tau is encoded by the *MAPT* gene, which is on chromosome 17q21 and contains 16 exons [35]. Alternative splicing of exons 2 and 3 results in variations in the N-terminal domain (NTD) of tau, where zero, one, or two inserts (0 N, 1 N, and 2 N) are present. Alternative splicing of exon 10 determines whether the microtubule-binding domain (MTBD) contains three or four microtubule-binding repeats (3R and 4R) [36,37,38]. The proline-rich domain (PRD) and the C-terminal domain (CTD) are identical in all six tau isoforms. The expression of tau isoforms is altered during brain development and differs between cell types and tissues [39,40,41].

Tau’s main function is to stabilize microtubules [1,2]. Binding of tau to microtubules is primarily mediated by the MTBD, with additional contributions from the flanking regions in the PRD and CTD [42,43]. The cryo-electron microscopy structure of the tau/microtubule complex demonstrates that tau adopts an extended conformation along the surface of the protofilament, which tethers tubulin dimers together [44]. Since the 4R tau isoforms contain one more binding repeat than the 3R isoforms, the 4R tau isoforms show a higher affinity for microtubules and promote microtubule assembly more efficiently [45]. In addition to stabilizing microtubules, the binding of tau to the microtubule surface also modulates the motility of motor proteins, thus regulating microtubule-dependent axonal transport [46].

Besides microtubules, tau interacts with numerous targets and participates in various biological processes through microtubule-independent protein–protein interactions [47,48,49,50]. Liu et al. studied the interactomes specific to tau isoforms with either 0, 1, or 2 N-terminal inserts [51]. They revealed preferential interactions for the three tau isoforms and found that many binding proteins are involved in synaptic signaling, energy metabolism, and cytoskeleton processing. Additionally, a segment of 11 amino acids (residues 18–28) in the NTD is specific to primate species. Stefanoska et al. found that this segment may mediate interactions involved in synaptic transmission and signaling [52]. SH3 domains are a group of protein modules that are crucial in various cellular pathways. They mediate protein–protein interactions by recognizing the proline-rich motifs on target proteins [53]. The PRD of tau contains seven proline-rich motifs, of which some can be recognized by the SH3 domains of the tyrosine kinase Fyn and membrane remodeling protein BIN1 [54,55,56]. The interaction between tau PRD and SH3 domain-containing proteins suggests that tau is involved in signal transduction pathways associated with neurodegeneration.

### 2.2. Structure of Tau Monomer

Tau is enriched with charged and polar residues, which prevents it from folding into a stable three-dimensional structure. Various experimental characterizations have demonstrated that tau is intrinsically disordered with dynamic secondary structure elements present in the PRD and MTBD [57,58]. While tau is disordered, dynamic intramolecular contacts drive compaction of tau conformation in solution. The NTD and CTD of tau are negatively charged, while the PRD and MTBD are positively charged. Attractive electrostatic interactions drive folding of the NTD and CTD over the PRD and MTBD [59]. As a result, the overall dimensions of tau are smaller than those of a random coil with similar length [60,61,62].

Conformational compaction has a role in modulating the functional state of tau. In a series of works, Joachimiak and collaborators demonstrated that different tau monomer groups can be separated by size exclusion chromatography [63,64,65]. Importantly, they found that tau monomer species adopting more compact conformations are in the seed-competent state [63,64,65]. Studies from the Goldsmith group also revealed that the tau monomer can reside in two groups of conformations with distinct extents of compaction and dynamics [66,67]. Notably, the transition between the more compact conformation group and the less compact conformation group is resistant to heat treatment and sensitive to guanidine hydrochloride treatment [63,66].

### 2.3. Tau Aggregation and Diseases

The identification of tau as the major component of fibrillar inclusions deposited in the brains of patients with neurodegenerative diseases, including Alzheimer’s disease, chronic traumatic encephalopathy, progressive supranuclear palsy, corticobasal degeneration, and frontotemporal lobar degeneration with tau pathology, has attracted significant attention. High-resolution structures of tau filaments isolated from the brain of a patient with Alzheimer’s disease were reported by Fitzpatrick et al. in 2017 [68]. Since then, substantial progress has been made in determining the structures of tau filaments in other tauopathies [69,70]. These studies revealed that tau filaments are formed by packing tau segments in β-strand conformations (primarily by residues 254–387 located in the MTBD) and tau can adopt different folded structures in the fibrillar state. Most importantly, while the folded structures of tau can be the same or different in distinct diseases, tau filaments from different individuals with the same disease show similar folds [69,71], indicating that a common mechanism and disease-specific modulation synergistically control tau fibrillation [72].

Despite extensive investigation, the pathogenesis of tau-mediated neurodegeneration remains unclear [73]. Some studies using animal models showed that hyperphosphorylated tau, either in monomeric or oligomeric states, is the toxic species in vivo [74,75,76]. However, the neuronal toxicity of NFTs is controversial. On the one hand, it has been suggested that the formation of insoluble NFTs is a protective mechanism to alleviate the toxic effects of soluble tau species and NFTs may act as an antioxidant against chronic oxidative stress [77,78]. On the other hand, there is a growing body of evidence that suggests a correlation between tau inclusion accumulation and cognitive impairment [79,80,81]. Additionally, fragmented tau fibrils can accelerate tau aggregation and promote tau spread [82,83,84,85]. Therefore, preventing tau aggregation and disassembling tau aggregates are potential therapeutic strategies targeting tau.

## 3. Liquid Condensates: A New Phase Linked to Tauopathies

Numerous studies over the past two decades have revealed that LLPS is a general mechanism that underlies the assembly of membraneless organelles and is closely linked to neurodegenerative diseases [86,87,88]. The LLPS capability of tau was identified in 2017 by the Zweckstetter group, Kosik and Han group, and Diez and Hyman group [21,24,89]. Since then, significant progress has been achieved, indicating the involvement of LLPS in the physiology and pathology of tau [16,17,18,19,90,91].

### 3.1. LLPS of Tau

**Figure 2:**
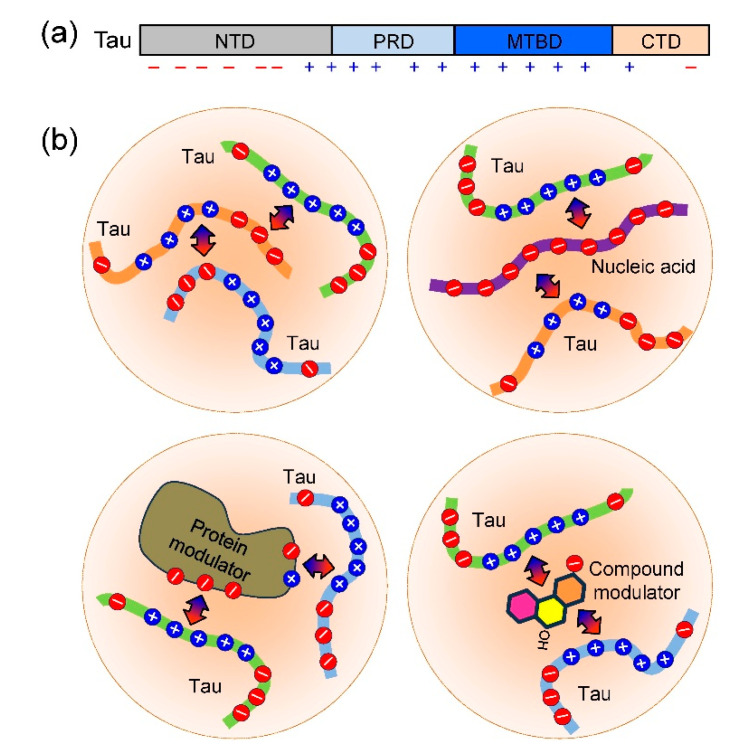
Schematic illustration of interactions mediating tau LLPS. (**a**) Distribution of charges on tau. (**b**) LLPS of tau is primarily driven by electrostatic interactions. When a co-factor (such as a nucleic acid, protein, or small compound molecule) is present, LLPS of tau can be modulated by electrostatic interactions, hydrophobic interactions, and hydrogen bonding.

When protein concentrations are above the saturation limit, they may undergo LLPS, which results in a protein-depleted dilute phase and a protein-enriched condensed phase. LLPS is driven by dynamic multivalent interactions, such as electrostatic interactions, π–π interactions, cation–π interactions, and hydrophobic interactions [92,93,94]. Tau is a polyelectrolyte with low contents of hydrophobic and aromatic residues. Therefore, nonspecific intermolecular electrostatic interactions between the negatively charged NTD and the positively charged PRD and MTBD drive tau LLPS under physiologically relevant conditions (Figure 2) [95,96]. Tau can also form heterotypic LLPS with nucleic acids, such as RNA [89] and DNA [97], and proteins, such as prion protein [98], α-synuclein [99], 14-3-3ζ [100], S100B [101], and TIA1 [102], where electrostatic interactions are expected to play a key role. As illustrated in fluorescence recovery after photobleaching experiments and confocal microscopy imaging, tau condensates display liquid-like properties, with tau molecules diffusing rapidly within condensates and small condensates fusing into larger ones. Tau molecules are significantly enriched in condensates. The concentration of tau in the condensed phase can be up to 100-fold higher than that in the dilute phase [25,103]. Moreover, the compact conformation of tau is opened in condensates, resulting in tau becoming more expanded [62,104]. Therefore, LLPS significantly alters the spatial distribution and conformational dynamics of tau, which could contribute to both the physiological function and pathological transition of tau.

### 3.2. Physiological Roles of Tau LLPS

Although in vivo evidence is still lacking, several in vitro studies suggest the physiological relevance of tau LLPS. Tau condensates modulate the assembly and function of microtubules. Tubulin is recruited into tau condensates, where microtubules nucleate and elongate, suggesting that tau condensates could function as non-centrosomal microtubule nucleation centers in neurons [21]. Besides tubulin, tau condensates recruit other biomolecules, including the plus-end tracking protein EB1 and RNA [105,106]. EB1 condenses at the microtubule plus-ends and regulates microtubule growth rate and catastrophe frequency [107]. Venkatramani et al. showed that EB1 impairs the ability of tau to promote microtubule assembly upon co-phase separation with tau [108]. It is possible that tau dissociates from microtubules due to competitive binding of EB1 to microtubules or EB1 prevents tau from binding to microtubules by interacting with the MTBD of tau [108]. Hochmair et al. showed that RNA and tubulin compete for the co-condensation with tau [106]. Their results indicated that the presence of RNA not only lowers the concentration of tubulin within tau condensates, but also impacts the morphology of microtubule bundles [106]. Tau condensates that form on the surface of microtubules have the potential to affect the function of microtubules. For example, tau condensates can function as a protective layer that regulates the activity of microtubule-severing enzymes and the movement of molecular motors on microtubules [22,23]. Tau condensate formation can also induce compaction of the microtubule lattice, thus biasing the conformational dynamics of tubulin within the lattice [109]. Additionally, tau forms transient nano-biomolecular condensates at the presynapse, where tau controls the mobility of recycling synaptic vesicles under the regulation of synaptic activity [110]. Furthermore, Abasi et al. demonstrated that tau promotes chromatin compaction and protects DNA from digestion through co-phase separation with DNA, nucleosomes, and heterochromatin protein 1α [97]. Therefore, tau phase separation in the nucleus may contribute to DNA protection and heterochromatin regulation.

### 3.3. Linking Tau LLPS to Tauopathies

While there is numerous evidence indicating that cellular compartmentalization via LLPS plays a crucial role in various biological processes [111,112,113,114], LLPS is also associated with many diseases, including neurodegenerative disorders [115], cancers [116], and infectious diseases [117].

Translocation of tau into the presynapse regulates the organization of synaptic vesicles [110]; however, localization of tau at the postsynapse may be correlated with synaptic dysfunction [118]. Shen et al. showed that tau undergoes co-phase separation with postsynaptic density scaffold proteins and induces dynamic arrest of postsynaptic density condensates in vitro, which may contribute to synaptic dysfunction due to impact on the clustering and trafficking of N-methyl-D-aspartate receptor into dendritic spines [119].

Fibrillar aggregation of tau is widely believed to be linked to tauopathies [72]. It turns out that LLPS accelerates tau aggregation [24,25,26,27]. Tau can be enriched by up to a 100 fold in the condensed phase compared to the dilute phase [25,103]. In addition, transient interactions within condensates induce expansion of tau conformation, which exposes the aggregation-prone hexapeptides located in the MTBD [62,104]. These two factors can work together to accelerate tau aggregation. Indeed, the lag phase of tau aggregation is significantly reduced and the rate of propagation is markedly increased under LLPS conditions [27,62]. Moreover, LLPS promotes the oligomerization of tau. Using purified recombinant proteins, Kanaan et al. showed that tau forms stable oligomers adopting pathogenic conformations when tau condensates become aged [25]. Interestingly, Lucas et al. found that when tau oligomers are recruited into tau condensates, they can catalyze the oligomerization of monomeric tau in condensates [120]. Recently, Soeda et al. discovered that the tau-CRY2olig fusion protein forms stable clusters in Neuro2a cells under blue light exposure [121]. They further demonstrated that these stable tau clusters are able to seed recombinant tau aggregation.

Many factors that promote tau aggregation have been found to modulate tau LLPS. Pro-aggregation mutations, such as P301L, P301S, A152T, and ΔK280, have been shown to increase the LLPS propensity of tau and/or decrease the dynamics of tau condensates in some studies [25,26,122,123]. PTMs, including phosphorylation, acetylation, and ubiquitination, are involved in the physiological functions and pathological processes of tau [124,125]. A growing body of evidence indicates that PTMs play a crucial role in modulating tau’s tendency to undergo phase separation [18,90]. The effect of a PTM on tau LLPS can be promotive, suppressive, or neutral, which depends on the site and type of modification [24,26,106,126,127,128,129,130,131,132]. Dyshomeostasis of metal ions is involved in the development of neurodegenerative diseases [133,134]. Particularly, several metal ions have been shown to accelerate tau aggregation and modulate tau phase separation [135,136,137,138].

Tau is recruited into stress granules under stress conditions. The stress granule nucleating protein T-cell intracellular antigen 1 (TIA1) associates with tau and facilitates the internalization of extracellular tau [139,140]. Importantly, TIA1 accelerates the liquid-to-solid transition of tau droplets and promotes the formation of tau oligomers [102,141,142]. Although the exact role of tau in stress granules is unclear, dysfunction of stress granules is implicated in the pathophysiology of tauopathies [143,144,145,146].

## 4. Combating Tauopathies in the Context of LLPS

**Figure 3:**
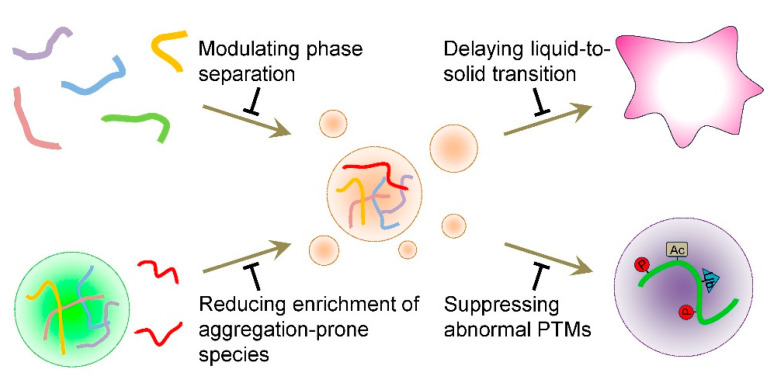
Therapeutic approaches for tau pathology in the context of LLPS. LLPS offers novel strategies for combating tauopathies, such as modulating tau phase separation, delaying the liquid-to-solid transition of tau condensates, reducing the enrichment of aggregation-prone species into tau condensates, and suppressing abnormal PTMs on tau. P: phosphorylation; Ac: acetylation; Ub: ubiquitination.

Significant efforts have been made to combat tauopathies, with the main objectives being to inhibit tau aggregation, clear tau aggregates, and attenuate abnormal PTMs on tau [28,29,147,148]. Previous strategies primarily target tau monomers, oligomers, and fibrils. The identification of liquid condensate as the fourth state of tau poses challenges to previous efforts but also offers new directions for future studies. LLPS is driven by dynamic multivalent interactions; thus, the formation of liquid condensates is readily modulated by various factors, including pH, salt, protein concentration, PTMs, small molecule compounds, and client macromolecules [92,149]. Below, we discuss the potential strategies for combating tauopathies in the context of LLPS (Figure 3).

### 4.1. Modulating Tau Condensate Formation

Because tau aggregation is accelerated in condensates, modulating tau LLPS under pathological conditions may delay the progression of tauopathies. A number of molecules, including macromolecules and small molecule compounds, have been found to interact with tau and significantly modulate tau’s LLPS propensity [90]. Among them, microtubule plus end-tracking protein EB1 [108], Ca^2+^-dependent chaperone S100B [101], peptidyl prolyl isomerase A (PPIA) [150], the main components of espresso coffee extract caffeine and genistein [151], the natural products shikonin [152] and myricetin [153], the synthetic derivative of curcumin C1 [154], and cyclic dipeptide-based small molecules [155] have been shown to impede tau phase separation. Some molecules prevent tau aggregation while augmenting tau phase separation, such as methylene blue (MB) [156], (−)-epigallocatechin-3-gallate [126], and suramin [157]. In addition, gallic acid [158] and tannic acid [159] are biphasic modulators of tau LLPS and inhibit the conversion of tau droplets into aggregates. The chemical structures of compounds that modulate tau LLPS differ significantly. However, hydrophobic moieties, negatively charged groups, and phenolic hydroxyl groups are usually present, suggesting that hydrophobic interactions, electrostatic interactions, and hydrogen bonding play a crucial role in modulating tau LLPS.

To elucidate the mechanisms underlying tau LLPS modulation, the Zweckstetter group and Gomes group used nuclear magnetic resonance spectroscopy to characterize the interactions between tau and PPIA [150], MB [156], and S100B [160], respectively. PPIA binding and PPIA-catalyzed cis/trans-isomerization of proline residues remodel the conformational ensemble of tau, resulting in the broadening of resonance signals throughout the entire sequence of tau [150]. Similarly, MB interacts with various regions of tau [156]. Unlike PPIA and MB, the S100B-affected regions of tau are located around the MTBD [160]. These results suggest that the interactions between tau and binding molecules are complicated and may vary significantly from one molecule to another. Nevertheless, our understanding of the regulatory mechanism is still limited because most studies are mainly focused on quantifying the effects of molecules on tau LLPS and the subsequent aggregation. In this context, it is unclear whether a molecule uses the same mechanism to inhibit tau aggregation within condensates as in the dilute bulk solution or not.

### 4.2. Delaying the Liquid-to-Solid Transition of Tau Condensates

Because LLPS plays a vital role in the physiological function of tau, dramatically enhancing or suppressing normal tau LLPS may be detrimental to disease treatment. In this context, delaying the liquid-to-solid transition (or aging) of tau condensates is preferred. The material properties of liquid condensates vary with time, and liquid-to-solid transition has been observed for many protein condensates [161,162,163,164,165]. Over time, intermolecular interactions strengthen and toxic tau oligomers form within the solidified condensates [25,120]. Disease-relevant mutations, PTMs, stress-induced protein-protein interactions, as well as RNA G-quadruplex could accelerate the liquid-to-solid transition of tau condensates [25,102,166,167]. Therefore, slowing down the liquid-to-solid transition of tau condensates could be a potential therapeutic strategy. Recently, Jonchhe at al. quantified the half-time of the liquid-to-solid transition of tau condensates in the presence of various small molecules [168]. Their findings suggested that the removal of water molecules from the hydration shell of tau molecules is an important factor for the liquid-to-solid transition of tau condensates. Importantly, they found that the osmolyte trimethylamine N-oxide is capable of efficiently slowing down the liquid-to-solid transition of tau condensates. While the mechanism is unclear, myricetin enhances the fluidity of tau condensates [153]. Besides small molecule compounds, proteins are also able to modulate the liquid-to-solid transition of tau condensates. For example, the tau-interacting proteins EB1 and protein disulfide isomerase enhance the fluidity of tau condensates and reduce tau aggregation [108,169]. Molecular chaperones are essential for protein homeostasis and can prevent the aggregation of many proteins related to neurodegenerative diseases [170,171]. Recently, several studies showed that heat-shock proteins, such as Hsp27 and Hsp40, are able to stabilize the liquid phase of FUS condensates and prevent amyloid aggregation [172,173]. A similar mechanism may be applied when Hsp40 and Hsp22 prevent tau aggregation [174,175,176].

### 4.3. Reducing the Concentration of Aggregation-Prone Species Within Tau Condensates

Protein aggregation normally proceeds via a nucleation-elongation mechanism [177]. Boyko et al. showed that the aggregation kinetics of tau under LLPS conditions differ from those under non-LLPS conditions [27]. Their findings indicated that nucleation is significantly increased and the lag phase becomes concentration-independent as the tau concentration in condensates is constant under LLPS conditions. Interestingly, they found that co-phase separation of tau with non-aggregating tau variants results in a reduction of aggregation due to a “dilution effect” [27]. Co-condensation of tau with the prion protein or α-synuclein enhances tau aggregation even in the absence of inducers [98,99]. Tau aggregation is a slow process and is usually induced with ionic inducers in in vitro experiments. On the contrary, prion protein and α-synuclein spontaneously aggregate without any required inducer. It has been found that α-synuclein fibrils can be recruited into tau condensates [178]. Therefore, it is possible that prion protein or α-synuclein fibrils formed within heteromolecular condensates seed the aggregation of tau. How to prevent the conversion of heteromolecular condensates into fibrils has not yet been studied. Based on the studies discussed above, reducing the concentration of aggregation-prone species could be a general strategy.

### 4.4. Suppressing Abnormal PTMs on Tau Within Condensates

The impact of PTMs on tau LLPS has been extensively studied [18,90,127]. Conversely, how LLPS affects tau PTM is not clear. A growing body of evidence shows that the rate of enzyme catalysis can be dramatically increased upon LLPS [179,180,181]. Specifically, the activity of several kinases has been found to increase under LLPS conditions compared to the bulk aqueous phase [182]. The intrinsically disordered CTD of RNA polymerase II (RNAPII) undergoes LLPS [183]. The cyclin-dependent kinase 7 (CDK7) associates with the surface of RNAPII CTD droplets, where it phosphorylates RNAPII CTD efficiently [183]. Tau can be phosphorylated by CDK2/cyclin A1, and the rates of phosphorylation are increased by several fold under LLPS conditions [184]. Unlike CDK7, CDK2 is highly enriched in the condensed phase [184]. cAMP-dependent protein kinase A (PKA) is another kinase contributing to tau phosphorylation. The regulatory subunit of PKA (RIα) undergoes LLPS on its own, producing liquid condensates enriched in cAMP and PKA activity [185]. In addition, tau ubiquitination can be facilitated under LLPS conditions. The E3 ubiquitin ligase TRIAD3A undergoes LLPS and recruits tau into condensates, where tau becomes ubiquitinated and forms aggregates [186]. Therefore, liquid condensates play a crucial role in regulating enzyme reaction rates by concentrating molecules and remodeling interactions [180,187,188,189,190,191]. The partition of a regulator (inhibitor or activator) between the condensed phase and the dilute phase can dramatically influence its activity. Furthermore, regulation can be achieved by modulating the composition of enzymatic condensates, which may bridge or interfere with interactions between the enzyme and the substrate.

## 5. Conclusions and Perspectives

Neurodegenerative diseases, including tauopathies, are a common threat worldwide. Despite enormous efforts and rapid advancements in the field, effective treatment remains lacking for these diseases. In this review, we provide an overview of the structure and phase transition of tau protein. In particular, we focus on the involvement of LLPS in the biology and pathology of tau. We discuss the potential of rational modulation of tau LLPS, including modulating the formation of tau condensates, delaying the liquid-to-solid transition of tau condensates, reducing the enrichment of aggregation-prone species into tau condensates, and suppressing abnormal PTMs on tau inside condensates, as a novel strategy for the development of therapeutic agents targeting tau pathologies in the future.

Although significant progress has been achieved in understanding tau phase transition in the past decade, there are still many open questions. For example, why do some tau aggregation inhibitors (e.g., MB and myricetin) exhibit opposite effects on tau condensate formation and liquid-to-solid transition? Are the interactions playing a crucial role in tau aggregation inhibition involved in tau LLPS modulation? How can we identify active molecules that modulate tau condensate formation and liquid-to-solid transition in a high-throughput manner? Answering these questions will deepen our understanding of the structure–activity relationship of tau phase transition modulators. Meanwhile, it is urgent to uncover the conformational changes of tau during phase transitions and the interactions between tau and modulators. Furthermore, the progression of tauopathies is associated with other biological processes, such as ferroptosis [192]. Synergistic effects could be achieved by targeting the LLPS of multiple targets [193,194].
